# A Defender of the Profession

**Published:** 1911-02-11

**Authors:** 


### A Defender of the Profession.

A year or two ago Mr. Rudyard Kipling delivered an address to the students of the Middlesex Hospital Medical School which brought much comfort to the medical profession. The thoroughly sympathetic spirit which informed the whole of that address, and the masterly prose in which it was composed, alike rendered the oration notable among the introductory addresses of the year, or indeed of any year. Under the title of "Doctors" it has been reprinted, and is listed among the other works of the author in the booksellers' catalogues. It did not, however, require the evidence of that admirable speech to prove that Mr. Kipling is a sincere and earnest friend of the medical profession; for ample indications of this fact can be found in his previous writings. We are not referring to the extraordinary knowledge of medical terminology which he displays; an uncanny familiarity with the technicalities of all the arts and sciences has always been one of his attributes, and it may be doubted whether he is as fluent in medical jargon as in that of engineering, for instance. It is rather the treatment of medical themes and medical characters which long ago established Mr. Kipling as a defender of the medical faith and of our professional honour. Mr. Barrie's sly digs at the foibles of medical men are always good humoured and never cause offence; but Mr. Kipling is almost more inclined to idealise us than to criticise.

In the latest volume by this pronounced medicophil—a collection of imaginary episodes from the lives of historical personages—there are two chapters of pronounced medical interest. In one of them the moral of the allegory will be readily drawn by cultured people in an England which is actually suffering from rat-plague; but in the other it is to be feared the author has veiled some of his allusions to the point of obscuring them altogether as far as the lay public is concerned. The book in question is *Rewards and Fairies;* incidentally it may be remarked that it contains a poem, entitled "If," which is certainly one of the finest ever written in our language.

In the tale entitled "A Doctor of Medicine" the author describes the work of a mediæval astrologer in staying the ravages of plague in an English village. Noticing the corn-chandler's shop as a centre of infection, and the immunity of the village smithy, his attention is further caught by the mortal sickness of the rats that swarm about the former place. He then conducts a crusade against the rats; not from any clear perception of the connection between human plague and rat plague, but because the "conjunctions," "trines," "antipathies," and so on of the planets indicated this as a means of stamping out the disease. The result of his efforts is complete success.

In "Brother Square-Toes" the allusions are less plain and patent. Briefly this is a story in which Læennec is shown to us perfecting the stethoscope while a prisoner of war in England. An English herbalist and witchmaster helps him to secure patients from among the yokels on whom to practise; and this same irregular practitioner has mastered the rudiments of breathing exercises, open windows, and, in fact, of the open-air treatment of tuberculosis. Whether we are meant to draw as an inference that the unqualified can sometimes teach the qualified a lesson, each reader must decide for himself. Certainly the ingenuity of these stories is remarkable, and shows that Mr. Kipling's interest in medicine has carried him a good deal more than skin deep in his researches into it.

